# Air-Liquid Interface Method To Study Epstein-Barr Virus Pathogenesis in Nasopharyngeal Epithelial Cells

**DOI:** 10.1128/mSphere.00152-18

**Published:** 2018-07-18

**Authors:** Elizabeth A. Caves, Sarah A. Cook, Nara Lee, Donna Stoltz, Simon Watkins, Kathy H. Y. Shair

**Affiliations:** a Cancer Virology Program, UPMC Hillman Cancer Center, University of Pittsburgh, Pittsburgh, Pennsylvania, USA; b Department of Microbiology and Molecular Genetics, University of Pittsburgh, Pittsburgh, Pennsylvania, USA; c Department of Cell Biology, University of Pittsburgh, Pittsburgh, Pennsylvania, USA; UNC-Chapel Hill

**Keywords:** air-liquid interface, Epstein-Barr virus, permissive infection

## Abstract

Epstein-Barr virus (EBV) is a ubiquitous gammaherpesvirus that establishes a latent reservoir in peripheral B-lymphocytes with sporadic reactivation. EBV also infects epithelial cells, predominantly resulting in a lytic infection, which may contribute to EBV transmission from saliva. In the nasopharynx, EBV infection can lead to the clonal expansion of a latently infected cell and the development of nasopharyngeal carcinoma (NPC). The mechanisms governing EBV pathogenesis in nasopharyngeal epithelium are largely unknown. An advanced understanding would depend on a physiologically relevant culture model of polarized airway epithelium. The recent application of the organotypic raft culture in keratinocytes has demonstrated great promise for the use of polarized cultures in the study of EBV permissive replication. In this study, the adaptation of an air-liquid interface (ALI) culture method using transwell membranes was explored in an EBV-infected NPC cell line. In the EBV-infected NPC HK1 cell line, ALI culture resulted in the completion of EBV reactivation, with global induction of the lytic cascade, replication of EBV genomes, and production of infectious progeny virus. We propose that the ALI culture method can be widely adopted as a physiologically relevant model to study EBV pathogenesis in polarized nasal epithelial cells.

**IMPORTANCE** Lifting adherent cells to the air-liquid interface (ALI) is a method conventionally used to culture airway epithelial cells into polarized apical and basolateral surfaces. Reactivation of Epstein-Barr virus (EBV) from monolayer epithelial cultures is sometimes abortive, which may be attributed to the lack of authentic reactivation triggers that occur in stratified epithelium *in vivo*. In the present work, the ALI culture method was applied to study EBV reactivation in nasopharyngeal epithelial cells. The ALI culture of an EBV-infected cell line yielded high titers and can be dissected by a variety of molecular virology assays that measure induction of the EBV lytic cascade and EBV genome replication and assembly. EBV infection of polarized cultures of primary epithelial cells can be challenging and can have variable efficiencies. However, the use of the ALI method with established EBV-infected cell lines offers a readily available and reproducible approach for the study of EBV permissive replication in polarized epithelia.

## Abstract (precis)

Lifting adherent cells to the air-liquid interface (ALI) is a method conventionally used to culture airway epithelial cells into polarized apical and basolateral surfaces. Reactivation of Epstein-Barr virus (EBV) from monolayer epithelial cultures is sometimes abortive, which may be attributed to the lack of authentic reactivation triggers that occur in stratified epithelium *in vivo*. In the present work, the ALI culture method was applied to study EBV reactivation in nasopharyngeal epithelial cells. The ALI culture of an EBV-infected cell line yielded high titers and can be dissected by a variety of molecular virology assays that measure induction of the EBV lytic cascade and EBV genome replication and assembly. EBV infection of polarized cultures of primary epithelial cells can be challenging and can have variable efficiencies. However, the use of the ALI method with established EBV-infected cell lines offers a readily available and reproducible approach for the study of EBV permissive replication in polarized epithelia.

## INTRODUCTION

Epstein-Barr virus (EBV) is a human pathogen that results in lifelong persistence, with more than 90% seroconversion in the adult population (1). EBV infects epithelial cells and B-lymphocytes, maintaining a latent reservoir in circulating memory B-cells with sporadic reactivation and transmission from oral secretions (2). Unlicensed replication in B-cells can manifest clinically as infectious mononucleosis, while productive replication in epithelial cells can be associated with immune suppression in AIDS patients in a disease known as oral hairy leukoplakia (OHL) (3). Latent infection is linked to all forms of EBV-associated cancers, including nasopharyngeal carcinoma (NPC) (1, 4, 5). EBV genomes are typically not integrated in the host cell, existing as circular episomal genomes in latently infected cells or as linear genomes in lytically replicating cells (6). During latency, the variable numbers of terminal repeats at the ends of the EBV genome fuse to produce uniquely sized BamHI-digested bands, which can be analyzed by Southern blotting in a termini assay to differentiate the states of infection (latent versus lytic and polyclonal versus monoclonal) (7). The state of EBV genomes in NPC tumors is latent and monoclonal, strongly supporting the hypothesis that EBV infection is present at the inception of neoplastic transformation (7, 8). Expression of the EBV oncoprotein latent membrane protein 1 (LMP1) in epithelial cells and rat-1 fibroblasts can promote oncogenic properties, including anchorage-independent growth and increased motility, and can also result in the formation of tumors in nude mice (8–12). Despite the association with oncogenic properties, it has been more difficult to elucidate the early events that lead to the establishment of latency and infection persistence in NPC (5, 13–15). The development of a robust *in vitro* method to mimic differentiation-induced lytic reactivation in polarized epithelia, in primary or immortalized airway epithelial cell lines, could significantly advance our interrogation of EBV pathogenesis in preneoplastic mechanisms.

The conventional method to reactivate EBV is by chemical induction with histone deacetylase (HDAC) inhibitors and protein kinase C inhibitors (12-O-tetradecanoylphorbol 13-acetate [TPA] and sodium butyrate) (6, 16). Alternatively, the lytic cascade can be triggered by transfecting the immediate early gene product zebra and late glycoprotein gB (6, 17). However, these methods do not recapitulate differentiation-induced reactivation and, depending on the cell line, can be abortive without production of progeny virus to appreciable titers (16, 18, 19). Moreover, not all cell lines are efficiently transfected and chemical induction inadvertently affects global host and viral epigenetics. The organotypic raft culture model established for studies in human papillomavirus (HPV) replication was recently applied to trigger EBV reactivation, resulting in the efficient production of infectious progeny virus that spreads in stratified primary keratinocytes (20). The organotypic raft culture can also be applied to the study of EBV infection in human telomerase reverse transcriptase (hTERT)-immortalized keratinocyte cell lines but is not always as robust a model for viral spread (21). One of the triumphs of the organotypic raft model for the study of EBV reactivation is that it is amenable to many standard DNA/RNA/protein molecular virology techniques evaluated either at the population level or at single-cell resolution by immunostaining and imaging methods (22). Nonetheless, the organotypic raft culture method selects for keratinocytes and is not yet a widely adopted technique. A method that can be applied to additional epithelial cell types and could be readily adopted for widespread use is the air-liquid interface (ALI) culture method, which is conventionally used to polarize primary airway epithelial cells of nasal or bronchial origin (23, 24).

**FIG 1:**
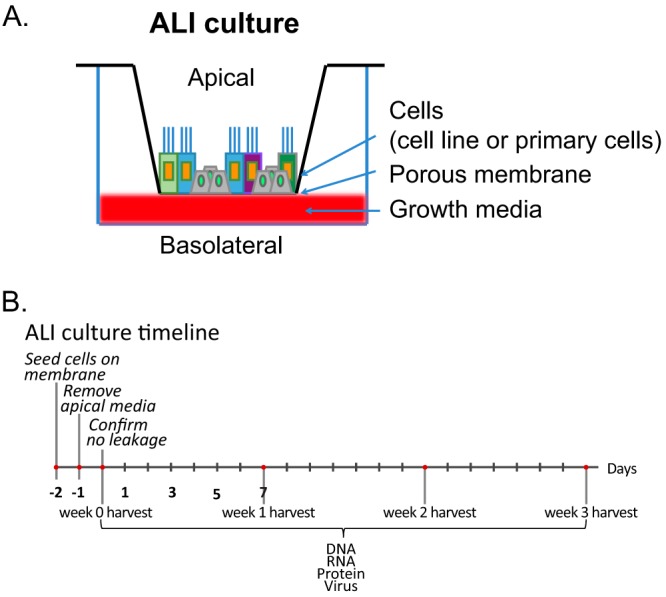
(A) Schematic and (B) timeline of the ALI culture model. Epithelial cells (primary or immortalized) are seeded on a collagen-coated transwell membrane. The polarized cells are fed from the basolateral surface.

The air-liquid interface (ALI) culture method establishes apical and basolateral surfaces by seeding cells on a collagen-coated (or equivalent extracellular matrix-coated) transwell membrane (25). Once an intact epithelium is established, the apical medium is removed and cells are fed through a porous membrane from the basolateral surface (Fig. 1A). Originally, the ALI method was used to establish pseudostratified cultures of primary airway epithelial cells with apical cilia and basolateral nuclei, which can preserve the diversity of cell types resembling native airway epithelium (23, 24). The ALI method can also be applied to immortalized cell lines (26, 27). ALI culture conditions have been routinely used for both primary and immortalized cells to study the pathogenesis of airway microbial pathogens (27, 28). Several studies have used the ALI method to define EBV infection parameters in polarized epithelia (26, 29, 30). However, only the organotypic raft culture has undeniably demonstrated that infection of stratified keratinocytes yields a permissive and productive infection (18, 20). While those few studies have been recognized as crucial and complementary, these intricate culture methods have yet to be widely adopted (31–33).

There are a limited number of authenticated NPC cell lines that continue to harbor latent EBV in culture (14, 34–36). In the present study, the ALI culture method was evaluated for induction of the EBV lytic cascade and production of progeny virus in EBV-infected nasopharyngeal and NPC-derived cell lines that are ordinarily latent in monolayer culture (37–39). The HK1 cell line, described as having originated from a differentiated NPC tumor biopsy specimen and infected with a recombinant (Akata) EBV strain, was amenable to ALI-induced culture conditions (38, 40). Lytic reactivation was then monitored by assessing lytic gene induction, EBV genome replication, and the production of infectious progeny virus. The results of this study demonstrate that the ALI method is proficient at reactivating EBV from the established HK1-EBV cell line, yielding high titers (~10^6^ packaged genome equivalents per ALI culture [1.12 cm^2^]) that are secreted and infectious, thus providing an alternative method to interrogate EBV permissive replication from polarized epithelia in an established NPC-derived cell line and allowing the elucidation of EBV pathogenesis in nasal epithelia.

## RESULTS AND DISCUSSION

### Induction of the EBV lytic cascade.

The NPC HK1 cell line is derived from a squamous carcinoma of the nasopharynx (40). The primary biopsy specimen was too small for EBV DNA analysis, but the outgrowing HK1 cell line did not show evidence of EBV infection as determined by staining for EBNA1 or by imaging of virus particles (40). The HK1-EBV cell line was established by *in vitro* infection of a recombinant EBV originating from the EBV Akata strain (38). In proliferating monolayer cultures, the EBV-infected HK1 cell line does not show induction of the lytic cascade and expresses a type II latency profile characterized by the expression of EBNA1, EBER1/2, LMP1, LMP2A, LMP2B, and BART transcripts (38). In this cell line, expression of lytic genes can be induced by the HDAC inhibitor suberoylanilide hydroxamic acid (SAHA) but is primarily abortive and does not yield progeny virus (16). Epithelial differentiation is a physiological trigger for EBV reactivation and can be induced by culturing cells at the air-liquid interface (32). Therefore, the HK1-EBV cell line was tested using the ALI epithelial cell polarization method for EBV reactivation and the timeline presented in Fig. 1B. Upon removal of apical media, harvest time points begin 1 day after lifting to the air-liquid interface (denoted week 0) and at weekly intervals for a total of 3 weeks. One additional hTERT-immortalized nasopharyngeal cell line (NP460hTERT-EBV) and one natively infected NPC cell line (C666-1) were also evaluated (37–39), but only the HK1-EBV cell line could be maintained under ALI growth conditions and could preserve an intact epithelium under polarized conditions (data not shown).

**FIG 2:**
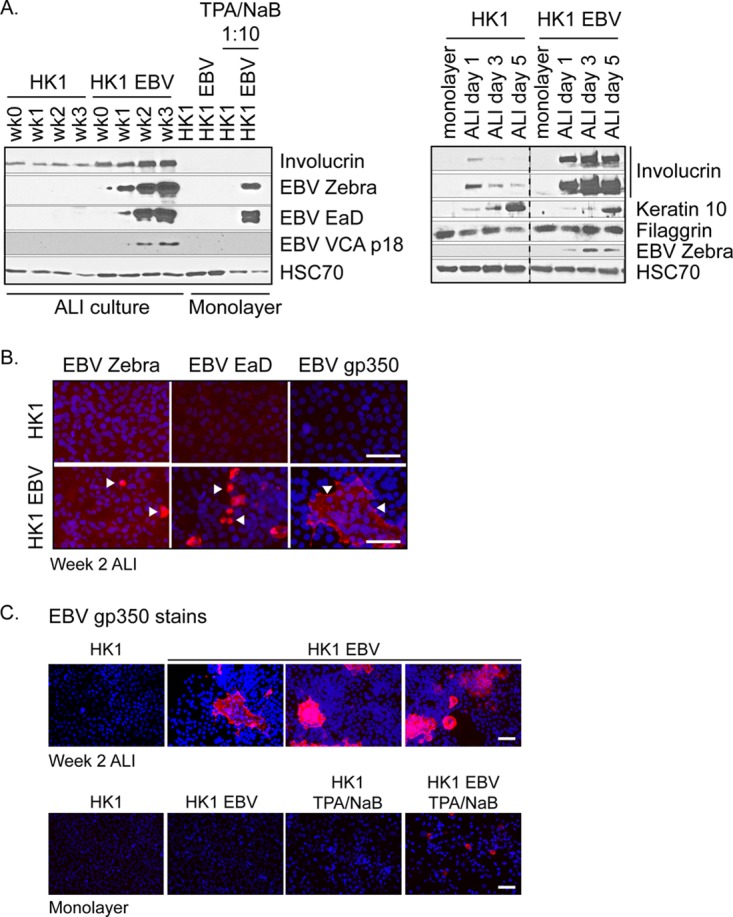
Induction of EBV lytic proteins in HK1-EBV ALI culture. (A) Immunoblot analysis for the expression of differentiation markers (involucrin, keratin 10, and processed filaggrin) and EBV lytic proteins (zebra, EaD, and VCA p18) in EBV-infected and uninfected ALI-cultured HK1 cell lines. Monolayer HK1 and HK1-EBV cultures with or without TPA/sodium butyrate induction were included for comparison and loaded at 1/10th of total protein lysates. Data for different antibodies are separated by horizontal white lines, and results from the same gel are grouped by a black border. Gels with intervening lanes that were cropped for labeling purposes are indicated by a vertical dotted line. (B) Immunofluorescence staining of HK1 and HK1-EBV cells at week 2 of ALI culture for the EBV lytic antigens zebra, EaD, and gp350 (red). (C) To reflect the frequency of reactivation, three representative fields of view are shown for the gp350 stain of HK1-EBV ALI cultures. For comparison, monolayer cultures were treated with TPA (200 nM) and sodium butyrate (5 mM) for 3 days to induce reactivation. Nuclei were counterstained with DAPI (blue). Scale bar, 50 µm. Images were acquired on an Olympus Provis epifluorescence microscope.

To assess the induction of EBV lytic proteins, HK1 uninfected and EBV-infected cell lysates were harvested at weekly intervals for 3 weeks after lifting to the air-liquid interface. Lysates were analyzed by immunoblotting for expression of immediate early protein zebra, early protein EaD, and the late viral capsid antigen (VCA) p18 protein, as well as the cellular differentiation markers involucrin, keratin 10, and filaggrin (Fig. 2A). Although the HK1 cell line is described as having originated from a differentiated squamous carcinoma biopsy, involucrin was not evident in monolayer culture but was robustly induced in ALI culture, consistent with the hypothesis that the ALI method triggers terminal differentiation. At early time points, involucrin and keratin 10 levels were induced as early as day 1, corresponding to induction of the EBV immediate early switch protein zebra (Fig. 2A). Processed filaggrin levels were not affected and were weak, requiring long exposures, which may reflect the low abundance and variable detection in nasal mucosa (41, 42). The induction of involucrin was strongest in EBV-infected HK1 cells compared to uninfected cells, but both the infected and uninfected cell lines displayed consistent induction of involucrin and keratin 10, supporting the idea that ALI culture triggers differentiation (Fig. 2A). In comparison to the undetectable levels in monolayer culture, the EBV zebra, EaD, and VCA p18 proteins were induced beginning at week 1 to week 2 post-ALI culture (Fig. 2A). Moreover, chemical induction by treatment with TPA and sodium butyrate in monolayer culture triggered lytic reactivation but did not result in the production of progeny virus as determined by the green Raji unit (GRU) assay (Fig. 2A; see also Table S1 in the supplemental material). Late lytic proteins, particularly glycoproteins, are notoriously difficult to probe by immunoblotting; therefore, expression of EBV zebra and EaD and of an additional late glycoprotein, gp350, was also analyzed by immunofluorescence staining. Nuclear staining of EBV zebra and EaD proteins and the cytoplasmic staining of gp350 further support the induction of the lytic cascade (Fig. 2B). Cells that stain positively for gp350 represent cells that have completed induction of the lytic cascade. Only sporadic cells stained positively for gp350 in monolayer culture when reactivated with TPA and sodium butyrate (Fig. 2C). However, gp350-positive cells were much more frequently detected in ALI-cultured cells (~25% to 40%) and were often detected as a group of cells in focal areas of staining (Fig. 2C). These data support the idea that the ALI culture method is more efficient at completing induction of the lytic cascade than chemical reactivation in monolayer culture.

10.1128/mSphere.00152-18.1 TABLE S1  EBV monolayer culture titers analyzed by the green Raji unit assay. 293 cells are efficiently transfected by zebra (Z) and gB constructs. HK1 and NP460 transfection efficiencies were suboptimal. TPA/sodium butyrate (40 nM/5 mM) treatment lasted up to 5 days. Download TABLE S1, DOCX file, 0.01 MB. Copyright © 2018 Caves et al. 2018 Caves et al. This content is distributed under the terms of the Creative Commons Attribution 4.0 International license.

**FIG 3:**
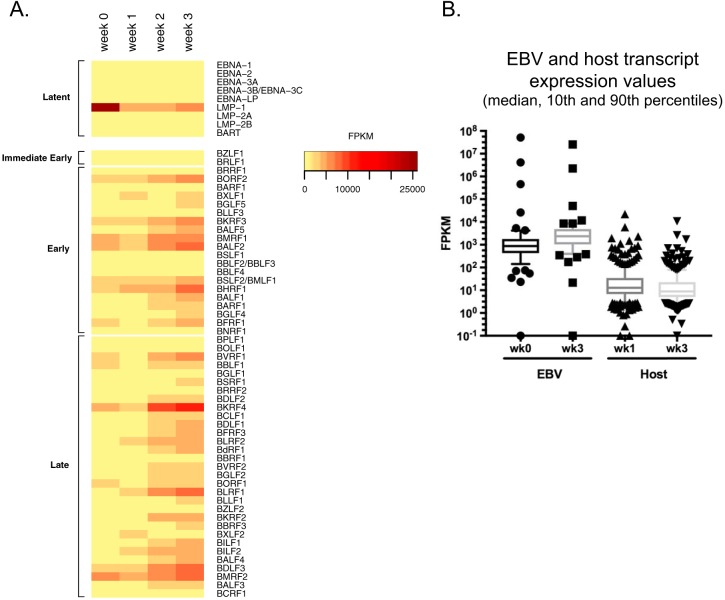
Induction of EBV lytic transcripts in HK1-EBV ALI culture. (A) RNA-seq profile of EBV latent and lytic transcripts collected from HK1-EBV cells at week 0 to week 3 of ALI culture. FPKM, fragments per kilobase of transcript per million mappable reads. (B) FPKM values of differentially regulated EBV and host transcripts with ≥2-fold change in HK1-EBV cells between week 3 and week 0 of ALI culture. Sequence reads were aligned separately to the EBV genome or the human genome and represented as FPKM, which takes into account the total number of mapped reads, and the data can be compared between week 0 and week 3 but cannot be compared between EBV and host values.

In addition to analysis of protein levels, RNA sequencing (RNA-seq) was performed to assess the global induction of EBV lytic transcripts. Of the 78 open reading frames (ORFs) annotated in the NCBI database, 65 were analyzed (Table S2) and 62 were represented as a heat map (Fig. 3A). Three ORFs, EBER1, EBER2, and BNLF2A, had extremely high numbers of reads and were not represented on the heat map but are illustrated in Table S2. The lytic genes were globally induced by week 2 to 3 in ALI culture, except for LMP1, LMP2B, EBER1/2, and BNLF2a, which showed a consistently decreasing trend overall (Fig. 3A). By comparison, host genes that were differentially regulated by at least 2-fold did not show an overall increase but a decrease in transcript levels which likely represented host shutoff (Fig. 3B; see also Table S3).

10.1128/mSphere.00152-18.2 TABLE S2  FPKM values of EBV transcripts. RNA-seq processed data from ALI-cultured HK1-EBV cells at weeks 0, 1, 2, and 3. *, high FPKM values not displayed in the heat map (Fig. 3B) are provided in this table. Download TABLE S2, XLSX file, 0.05 MB. Copyright © 2018 Caves et al. 2018 Caves et al. This content is distributed under the terms of the Creative Commons Attribution 4.0 International license.

10.1128/mSphere.00152-18.3 TABLE S3  FPKM values of differentially regulated host transcripts. Transcripts with ≥2-fold change between week 3 and week 0 of the HK1-EBV ALI culture are displayed. Upregulated transcripts are not highlighted; highlighted transcripts are downregulated at week 3. Download TABLE S3, XLSX file, 0.03 MB. Copyright © 2018 Caves et al. 2018 Caves et al. This content is distributed under the terms of the Creative Commons Attribution 4.0 International license.

The majority of EBV transcripts are expressed at up to 10^4^ fragments per kilobase of transcript per million mappable reads (FPKM), but the abundant expression of EBERs at 10^6^ to 10^7^ FPKM is intriguing (Table S2). EBERs are noncoding nuclear transcripts that are abundantly expressed in all EBV-associated cancers and latencies, with up to 10^6^ copies per cell in B-cell infections (43). Therefore, the abundant expression of EBERs is exploited for the diagnosis of EBV-associated cancers and diseases by EBER *in situ* hybridization (EBER-ISH) (44). One understanding of EBER function is that EBER2 can serve as a ribonucleoprotein complex to recruit DNA binding proteins to the terminal repeats on the EBV genome (45). The only disease pathology known to be associated with permissive replication in epithelial infection is the AIDS-associated nonmalignant lesion known as OHL that manifests on tongue and gingival tissues (3, 32, 44). Paradoxically, EBER transcripts are suppressed and sometimes not detected in OHL formalin-fixed paraffin-embedded tissues, which has called into question the function of EBERs during EBV reactivation (46, 47). The diagnosis of OHL may require secondary confirmation by staining for zebra (48). The presence of highly abundant EBER transcripts detected in the ALI culture method is consistent with the recent discovery that EBER transcripts are also detected by EBER-ISH throughout the layers of the stratified epithelium in organotypic raft cultures, which would suggest that EBERs are indeed expressed during EBV reactivation in the differentiated apical layers (20). These findings, combined with observations from the present study, would seem to imply that the suppression of EBERs may be unique to OHL.

### EBV genome replication and amplification.

**FIG 4:**
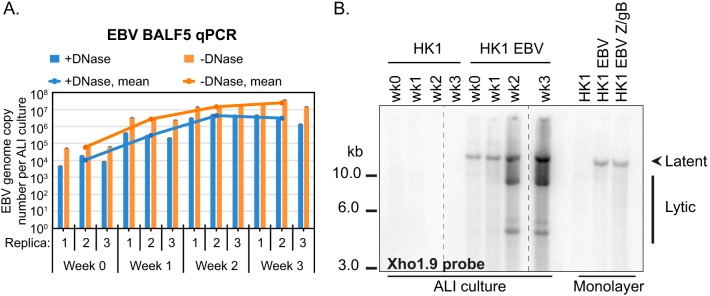
Measuring EBV genome amplification and replication in HK1-EBV ALI culture. (A) Quantitative PCR measuring EBV genome equivalents in HK1 ALI-cultured EBV-infected cells. The averaged value was calculated from three biological triplicates, and each biological triplicate value and each error were determined from three technical replicates. Encapsidated genomes are DNase resistant (+DNase), and total (encapsidated and nonencapsidated) genomes were determined in the absence of DNase treatment (-DNase). (B) Termini assay of ALI-cultured HK1-EBV cells in comparison to zebra (Z)- and gB-transfected monolayer-cultured cells. Shown is a Southern blot of BamHI-digested genomic DNA hybridized to a random-primed and radiolabeled Xho1.9 fragment DNA probe located on the BamHI-digested fragment leftward of the EBV terminal repeats. Data are displayed from the same blot and exposure time. Intervening lanes were cropped for labeling purposes and are demarcated by a vertical dotted line.

EBV genome amplification and replication were assessed by quantitative PCR (qPCR) and Southern blot analyses. Encapsidated genomes from DNase-resistant Hirt-purified extrachromosomal DNA were measured by qPCR. Total (encapsidated and nonencapsidated) EBV genomes increased more than 2 log_10_, beginning at 6.1 × 10^4^ EBV genomes per ALI culture at week 0 and reaching 2.5 × 10^7^ EBV genomes per ALI culture at week 3 (Fig. 4A). The majority of genomes were encapsidated with less than 1 log_10_ difference, starting at 1.1 × 10^4^ EBV genomes per ALI culture at week 0 and reaching 3.0 × 10^6^ EBV genomes per ALI culture at week 3 (Fig. 4A).

EBV latent genomes are circular and are detected as a single band (>10 kb) by Southern blotting (7). Replicating EBV genomes are linear and differ by 500-bp increments corresponding to the number of tandem terminal repeats (7, 48). At week 0 and week 1, a single band indicative of latent genomes greater than 10 kb in size was detected (Fig. 4B). In comparison at weeks 2 and 3, multiple bands smaller than 10 kb in size were detected with an overall increase in hybridization intensity for all detected bands, indicative of amplified replicating genomes (Fig. 4B). However, only one band associated with latent genomes was detected in monolayer culture and there was no appreciable increase in band intensity, indicating that EBV genomes were not replicating as efficiently as in ALI culture (Fig. 4B).

### Production of EBV progeny virus.

**FIG 5:**
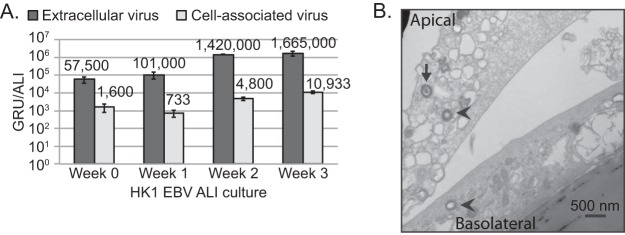
Production of infectious virus in HK1-EBV ALI culture. (A) Green Raji unit (GRU) titers measuring numbers of infectious units were determined for extracellular and cell-associated virus from HK1-EBV ALI cultures. Error bars were calculated from four replicate green Raji titers. Analyses of titers from week 2 were repeated in three biological replicates with the following values: 1.15 × 10^6^ ± 3.8 × 10^5^ GRU/ml (extracellular) and 4,000 ± 949 GRU/ml (cell associated). (B) Transmission electron micrograph (TEM) of HK1-EBV cells at week 2 of ALI culture. Herpesvirus-like particles (~250 nm) were visually identified by an independent microscopist. A packaged mature virion (arrow) and unpackaged immature virions (arrowhead) are illustrated.

Production of EBV progeny virus was analyzed by determining the titers of infectious virus with the GRU assay and by imaging with transmission electron microscopy (TEM). Raji cells are a Burkitt lymphoma cell line that can be readily superinfected with EBV, but the endogenous EBV genome is truncated and will not replicate, thus enabling the determination of the titers of ALI culture-derived green fluorescent protein (GFP)-expressing virus (49, 50). Overall titers, including extracellular and cell-associated virus titers, increased from week 0 to week 3. Extracellular virus titers were consistently 2 log_10_ higher than cell-associated virus titers, as would be expected from immature cell-associated virions. By week 3, there was a total value of 1,675,933 GRUs per ALI, demonstrating that abundant and infectious EBV virions were produced (Fig. 5A). This increase at week 3 was ~30-fold higher than the values measured at week 0, when production of progeny virus began. In comparison to the ALI culture results, no infectious units were measured in uninduced HK1-EBV monolayer-cultured cells (Table S1). Despite the expected loss of sample from Hirt purification, at week 2 to 3 of ALI culture there were 3.0 × 10^6^ to 4.3 × 10^6^ DNase-resistant encapsidated EBV genomes per ALI culture (1.12 cm^2^), which corresponds to 1.425 × 10^6^ to 1.676 × 10^6^ total GRUs per ALI culture (Fig. 4A and 5A). These data support the idea that the majority of encapsidated virions produced from ALI culture are indeed infectious.

HK1-EBV ALI cultures harvested at week 2 were imaged for evidence of EBV virions by TEM and assessed by an independent microscopist. Although extracellular virions are largely washed away during TEM processing, intracellular virions can be captured. The TEM images show an encapsidated herpesvirus-like virion of 200 to 250 nm and two immature virions in the cytoplasm (Fig. 5B). Additionally, 1 to 2 cells layers were observed in the TEM images, illustrating that the HK1-EBV ALI cultures were polarized but not necessarily stratified (Fig. 5B), which is representative of the pseudostratified differentiated ALI cultures of primary nasal epithelia (24). These data support the idea that the ALI method is a robust method for inducing fulminant EBV lytic reactivation without the need for chemical induction. The outcome of this study from an NPC-derived cell line perhaps appears to contradict the observation of latent infection in NPC tumors (7). Given that there was no evidence of EBV infection in the original NPC biopsy specimen from which HK1 cells were derived, it is plausible that the outcome of infection might differ from that seen with NPC tumor cells that have evolved to sustain latent infection *in vivo* (40). Despite the paucity of authenticated NPC cell lines, it would be interesting to test additional NPC cell lines that originate from tumors with confirmed EBV positivity (34). Furthermore, since HK1 cells can be recombinantly modified and stably selected, the HK1-EBV ALI culture model offers the potential to test host and viral factors which may predispose a neoplastic cell to EBV latent infection (25, 38). The ALI polarization culture method has been established for primary nasal epithelial cells (24). Furthermore, EBV infection as defined by the presence of latent or lytic markers is not detected in nasal biopsy specimens of histologically normal areas, likely reflecting rare or focal airway epithelial infections in asymptomatic carriers (51, 52). Due to the absence of detection of EBV infection *in situ* from nasal biopsy specimens of healthy tissue, in theory it is possible to study *de novo* EBV infection in ALI-cultured primary nasal epithelial cells. Ultimately, the creation of an EBV infection model and analytical methods to study (widespread or focal) infection in polarized ALI cultures of primary nasal epithelial cells would be the most relevant system to evaluate EBV *de novo* infection in mucociliated epithelia (53). Given the limited availability of primary nasal cells, the ALI culture method described for the HK1-EBV cell line thus offers a widely accessible alternative for the study of EBV permissive replication in polarized epithelia.

To date, there have been a few exemplar studies that have explored the effect of EBV proteins on differentiation, or differentiation-induced effects on EBV infection (18, 54–56). These experimental models include calcium-induced differentiation, suspension of cells in methylcellulose, and organotypic raft cultures (57). Keratinocytes grown in monolayer can be induced to differentiate by high levels of calcium, which has been applied to EBV studies by treatment with fetal bovine serum (FBS) and/or by the direct addition of calcium (18, 21, 56). A second method to differentiate keratinocytes is by suspending cells in methylcellulose, which has been applied to the study of EBV-infected cells and of the effect of the EBV latent protein, LMP2A, on differentiation (21, 57). The methylcellulose method can recapitulate differentiation in a three-dimensional (3D) culture model, but the analysis of these cells in a semisolid matrix is often limited to protein lysates harvested from the cell population (21, 57). Organotypic rafts provide single-cell resolution by formalin-fixed paraffin-embedding (FFPE) sectioning and staining and serve as a robust model for culturing fully stratified squamous keratinocytes, but the keratinocyte-enriching media used to establish organotypic rafts may not culture all NPC cell types (22). In principle, organotypic rafts, originally established for the study of EBV infection in oral keratinocytes, can also be developed for nasal keratinocytes (18, 20). The ALI culture method ultimately extends the application of polarization to other epithelial cell types. When applied to primary cells, ALI culture preserves the diversity of cell types represented in airway epithelium, including ciliated, mucosecretory, and basal cells (22). The ALI method described in this study for an NPC cell line (HK1) permits the analysis of protein expression at the population level but also with single-cell resolution (Fig. 2), demonstrating that the ALI culture method, as in the use of organotypic rafts, is amenable to a variety of molecular virology analytical techniques, including those involving DNA, RNA, protein, imaging, and the collection of infectious virus. Therefore, ALI culture is a method complementary to the use of organotypic rafts that can model EBV infection for the additional cell types represented in polarized airway epithelium.

## MATERIALS AND METHODS

### Setup of the ALI culture.

The overall setup of the ALI culture for the HK1 cell line has been previously described (25). Detailed experimental parameters are as follows. A total of 0.5 × 10^6^ cells are seeded on thin-coat collagen-coated polyester membrane transwells (Corning) (0.4 µm pore size, 12-mm diameter) in 0.5 ml apical medium. Cells are fed from the basolateral surface with 1 ml of media used for the propagation of HK1 cells (RPMI 1640 supplemented with 10% fetal bovine serum). Thin-coat collagen is prepared by diluting 0.1 mg/ml stock of fibrous collagen (IV) from human placenta (Sigma) in water supplemented with 200 µl glacial acetic acid (100% [vol/vol]), with rocking overnight at 4°C to facilitate solubility. The stock collagen solution is sterile filtered and further diluted 1:10 in 0.1 M Na_2_CO_3_ to a working concentration. Collagen stocks can be aliquoted and frozen at −20°C, but diluted stocks should be made fresh. For a 12-well plate, 250 µl of the collagen working stock is used to coat the apical surface of the transwell at 37°C for a minimum of 30 min and up to 1 h. Before the cells are seeded, the collagen solution is aspirated and gently rinsed once with phosphate-buffered saline (PBS). At 1 to 2 days postseeding, a confluent layer should form. The apical medium is removed, and the basolateral medium is replenished. Beginning as early as the next day, a successful ALI culture is established when there is no medium leakage into the apical surface, and that day is noted as day zero. Cells at the ALI are fed twice a week from the basolateral compartment and cultured for up to 3 additional weeks. The uninfected cells should have minimal apical leakage over the 3-week period and should be gently removed at every feeding. The ALI culture of EBV-infected cells increasingly disrupts the monolayer, and apical leakage is expected to increase over the 3-week period, but the integrity of the monolayer should still be apparent by visual inspection with phase microscopy.

### Analysis of EBV protein induction by immunoblotting, immunofluorescence, and RNA-seq.

The subsequent procedures are described for a 12-well plate transwell configuration. At weekly intervals, protein lysates were harvested. The basolateral media and apical leakage were removed. Cells were gently rinsed once in PBS and lysed in 50 µl of radioimmunoprecipitation assay (RIPA) buffer supplemented with 1 mM phenylmethylsulfonyl fluoride, 2 mM activated sodium orthovanadate, and a 1:100 dilution of protease and phosphate inhibitor cocktails (Sigma). The cells were lysed directly in the transwell by rocking at 4°C for 30 min followed by scraping and were clarified at ≥20,000 × *g* for 5 min at 4°C. Protein concentration, separation by SDS-PAGE, and immunoblotting was performed as previously described (25). Antibodies for immunoblotting were purchased from Santa Cruz Biotechnologies (EBV zebra clone BZ1; EaD clone 0261; filaggrin clone AKH1; HSC70 clone B-6), Thermo Scientific (EBV VCA p18; keratin 10 clone Ab-2), and Sigma (involucrin clone SY5).

For immunofluorescence staining, cells were fixed in 4% paraformaldehyde at room temperature and punched out of the transwell with the lid of a cryovial (or by the use of the top end of a P1000 tip cut to the size of the transwell). The use of a scalpel is not recommended since this can wrinkle the membrane and create an uneven surface for imaging. Cells were permeabilized with 0.1% Triton X-100–PBS for 5 min and washed in wash buffer (0.05% Tween 20–PBS) followed by blocking with 1% bovine serum albumin–PBS for 1 h at room temperature. Cells were probed with primary antibody diluted in blocking buffer and incubated overnight at 4°C in a humidified chamber. Following three rinses in wash buffer, cells were incubated with secondary antibody diluted in PBS for 1 h at room temperature. For EBV zebra (clone BZ1), EaD (clone 0261), and gp350 (clone 0221) stains, all primary antibodies (Santa Cruz Biotechnology) were diluted 1:100. EBV zebra and EaD were detected with Cy3-conjugated donkey anti-mouse IgG secondary antibody (Jackson ImmunoResearch) at 3.75 µg/ml. EBV gp350 was detected with biotin-conjugated donkey anti-mouse IgG diluted at 3 µg/ml and rhodamine red-conjugated streptavidin at 0.78 µg/ml (Jackson ImmunoResearch). Cells were counterstained in DAPI (4′,6-diamidino-2-phenylindole) and mounted in aqueous mounting media. Epifluorescence images were acquired on an Olympus Provis microscope with Q-Capture software. All images were adjusted for equal levels of intensity and brightness.

Total RNA was isolated using Trizol (Invitrogen) from HK1-EBV cells after induction of the lytic cycle at the indicated time points (week 0, week 1, week 2, and week 3). Briefly, cells were lysed in 1 ml of Trizol, purified with chloroform, precipitated with isopropanol followed by two washes of 75% ethanol, and resuspended in water. DNase-treated RNA (Turbo DNA-free kit; Ambion) was submitted to the Health Sciences Sequencing Core at the Children’s Hospital of Pittsburgh for quality assurance/quality control (QA/QC) analysis and quantitation on an Agilent TapeStation and Qubit. rRNA is susceptible to degradation during EBV reactivation; therefore, the expected RNA integrity number (RIN) can range from 2.4 to 5.5, with an expected total RNA yield in the range of 0.2 to 7 µg per ALI culture sample, decreasing as reactivation progresses. A more accurate measure of transcript integrity should be determined by the percentage of nucleotide fragments larger than 200 bp or by Northern blotting for intact EBV transcripts such as the abundant nonpolyadenylated EBERs.

Following QA/QC analysis, DNase-treated RNA samples were subjected to rRNA depletion using a Ribo-Zero Epidemiology kit (Illumina) and were converted into a deep sequencing library using Illumina’s Truseq stranded total RNA kit. Deep sequencing was performed on a NextSeq500 platform using a Mid-output kit and a 2 × 75-cycle paired-end run. We obtained 47.1, 49.4, 46.9, and 47.7 million reads for samples at week 0, week 1, week 2, and week 3, respectively. Sequence reads were trimmed by 10 nucleotides based on read quality and subsequently aligned to the EBV Akata genome (GenBank accession number KC207813.1) or to human reference genome hg38 using TopHat2 (58) and the options “—no-novel-juncs -r 300—library-type fr-firststrand.” Among the total reads, 0.4%, 0.2%, 0.8%, and 0.8% aligned to the EBV genome for sample week 0, week 1, week 2, and week 3, respectively; the alignment rates for hg38 were 82.7%, 84.1%, 80.0%, and 82.2%, respectively.

To generate a heat map of EBV transcript abundance, Cufflinks (59) was used to calculate FPKM values of EBV genes, which were then applied to the heatmap.2 tool in the gplots package (60). For analyzing differential host gene expression, the algorithm CuffDiff2 (61) was used to compare week 0 transcriptomes and week 3 transcriptomes with the options “—library-type fr-firststrand—compatible-hits-norm—max-bundle-frags 1000000000000.” Table S3 in the supplemental material includes data from host genes that exhibited a 2-fold change between the week 0 and week 3 samples.

### Analysis of EBV DNA genome replication by qPCR and Southern blotting.

Cells were harvested by scraping in 100 µl of PBS followed by three cycles of rapid freezing-thawing to release virions. Cellular debris was pelleted at ≥20,000 × *g* for 5 min, and the supernatant was harvested and split for subsequent experiments performed with or without DNase treatment. Half of the sample was treated with a Turbo DNA-free kit (Ambion) to isolate encapsidated genomes. Both the samples subjected to DNase treatment and those left untreated were subjected to Hirt purification for isolation of extrachromosomal EBV genomes (22). For every 25-µl sample, 415 µl Hirt buffer, 5 µl of 20 mg/ml proteinase K, and 25 µl of 10% SDS were added and incubated for 2 h at 37°C. This was followed by phenol-chloroform extraction (470 µl phenol-chloroform-isoamyl alcohol [25:24:1]) and separation into the aqueous phase by spinning at ≥20,000 × *g* for 10 min, followed by washing the aqueous layer with 470 µl of chloroform and collecting the aqueous layer by spinning at ≥20,000 × *g* for 10 min. DNA was precipitated with 0.1× 3 M sodium acetate and 2.5× 100% ethanol and was incubated overnight at −20°C. To pellet DNA, samples were spun at ≥20,000 × *g* for 10 min at 4°C, washed with 300 µl 70% ethanol, and pelleted again at ≥20,000 × *g* for 10 min at 4°C. The DNA pellet was air dried and resuspended in 12.5 µl of water. Of note, it would also be possible to perform the qPCR procedure using column-purified total genomic DNA and normalization to a cellular target but the procedure would need to be empirically optimized for sensitivity and template quantity.

For qPCR, 2 µl of the Hirt-purified DNA was analyzed by the Sybr green absolute quantitation method. A standard curve was generated with 10^0^ to 10^9^ copies per reaction using a BALF5 plasmid (7,782 bp) as the template (62). Reaction mixtures were assembled with a Maxima SYBR green qPCR master mix kit (Thermo Scientific) with 0.3 µM of each primer, using recommended cycle conditions with ROX as a passive reference dye in a total reaction volume of 10 µl. Primer sequences are as follows: for qBALF5-F, 5′ GAGCGATCTTGGCAATCTCT 3′; for qBALF5-R, 5′ TGGTCATGGATCTGCTAAACC 3′. Reactions were amplified on a StepOnePlus instrument (Applied Biosystems) and analyzed with StepOne software (v2.3). Average values and standard deviations were calculated from triplicate technical replicas and three independent experiments. Water and uninfected HK1 ALI culture cells from corresponding weeks were used as negative controls. End values were represented as BALF5 copy number per ALI culture.

For Southern blotting, total genomic DNA was harvested from Trizol-lysed cells or could also be harvested by column purification (GeneJET genomic DNA purification kit with RNase treatment; Thermo Scientific). Total DNA was isolated from the nonaqueous phase according to manufacturer’s instructions (Invitrogen). The DNA harvested from each ALI culture was digested with BamHI, ethanol precipitated in the presence of 5 to 10 µg of glycogen, and loaded on an agarose gel. A linearized Xho1a (1.9-kb) probe was generated by random-prime synthesis, radiolabeled, and hybridized as previously described (63).

### Measuring EBV titers.

Residual media on the apical side were collected. Intracellular and extracellular virus was harvested by scraping cells in 100 µl of RPMI media followed by an additional 50-µl rinse and was collected to reach a total volume of 200 µl. The sample was clarified by spinning at 500 × *g* for 5 min, and the supernatant was collected and stored at 4°C as extracellular virus. The cell pellet was resuspended in 200 µl of RPMI media followed by three cycles of rapid freezing-thawing to release intracellular virus. Disrupted cells were spun down at 500 × *g* for 5 min, and the supernatant was collected and stored at 4°C as cell-associated virus.

Infectious titer was determined by the green Raji Unit (GRU) assay (50). In a 96-well plate, 90 µl of RPMI medium was added to 10 µl of virus stock and titrated in 10-fold serial dilutions (10^0^ to 10^9^). Ten thousand Raji cells in 10 µl media were added to each well and, after a 48-h inoculation period, reactivated with 200 nM TPA and 5 mM sodium butyrate overnight. A fluorescence microscope was used to count the number of GFP-positive cells in each well to determine the number of GRU per milliliter.

### TEM.

HK1 and HK1-EBV ALI cultures harvested at week 2 were fixed in cold 2.5% glutaraldehyde–0.01 M PBS. Specimens were rinsed in PBS, postfixed in 1% osmium tetroxide with 1% potassium ferricyanide, rinsed in PBS, dehydrated through a graded ethanol series, and embedded in Poly/Bed 812 (Luft formulations). Semithin (300-nm-thick) sections were cut on a Leica Reichart Ultracut ultramicrotome, stained with 0.5% toluidine blue–1% sodium borate, and examined under the light microscope. Ultrathin sections (65 nm thick) were stained with uranyl acetate and Reynold’s lead citrate and examined on a JEOL 1011 transmission electron microscope with a side-mount AMT 2-k digital camera (Advanced Microscopy Techniques).
