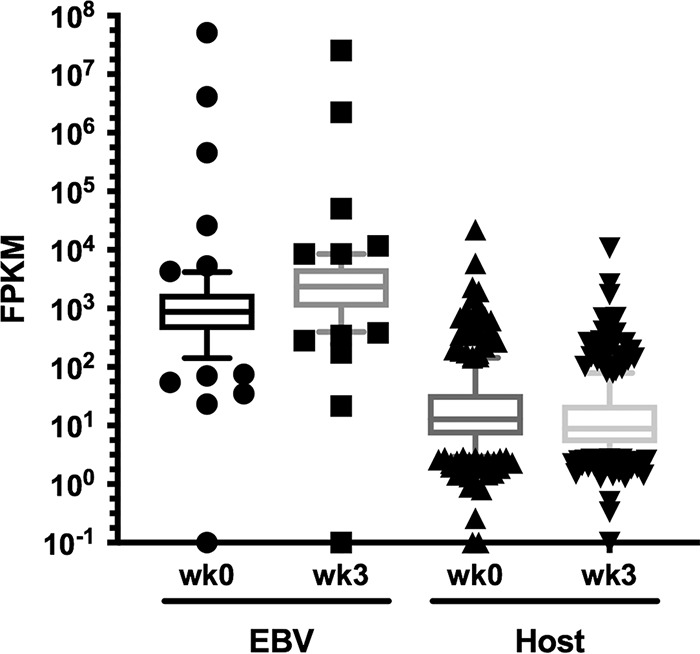# Erratum for Caves et al., “Air-Liquid Interface Method To Study Epstein-Barr Virus Pathogenesis in Nasopharyngeal Epithelial Cells”

**DOI:** 10.1128/mSphere.00247-19

**Published:** 2019-04-17

**Authors:** Elizabeth A. Caves, Sarah A. Cook, Nara Lee, Donna Stoltz, Simon Watkins, Kathy H. Y. Shair

**Affiliations:** a Cancer Virology Program, UPMC Hillman Cancer Center, University of Pittsburgh, Pittsburgh, Pennsylvania, USA; b Department of Microbiology and Molecular Genetics, University of Pittsburgh, Pittsburgh, Pennsylvania, USA; c Department of Cell Biology, University of Pittsburgh, Pittsburgh, Pennsylvania, USA

## ERRATUM

Volume 3, no. 4, e00152-18, 2018, https://doi.org/10.1128/mSphere.00152-18. In Fig. 3B, one of the series for the FPKM values in the host transcriptome was mislabeled week 1 (wk1); it should instead be week 0 (wk0). The corrected graph is shown below.

**FIG 3B:**