# Supplementary material for: Air-Liquid Interface Method To Study Epstein-Barr Virus Pathogenesis in Nasopharyngeal Epithelial Cells
Source: mSphere. 2018 Jul 18;3(4):e00152-18. doi: 10.1128/mSphere.00152-18 (PMC6052337; doi:10.1128/mSphere.00152-18)
Supplement: TABLE S1 [file sph004182602st1.docx]

| **Cell line** | **Reactivation Condition** | **GRU/ml** |
| --- | --- | --- |
| 293 EBV | None | 0 |
| 293 EBV | Z/gB transfection | 3.90*10^8^ |
| HK1 EBV | None | 0 |
| HK1 EBV | TPA and sodium butyrate | 510 |
| HK1 EBV | Z/gB transfection | 1.32*10^5^ |
| NP460 EBV | None | 0 |
| NP460 EBV | TPA and sodium butyrate | 0 |
| NP460 EBV | Z/gB transfection | 0 |
